# Associations of exercise frequency and type with trajectories of daily activity limitations among older adults in the UK

**DOI:** 10.1186/s12916-026-05058-6

**Published:** 2026-07-10

**Authors:** Hong Ding, Wen Wang

**Affiliations:** 1 College of Humanities and Health Sciences, Bengbu Medical University, Bengbu, 233030 China; 2 Institute for Social and Economic Research, University of Essex, Colchester, CO4 3SQ UK

**Keywords:** Exercise frequency, Exercise type, Older adults, Functional limitations, Longitudinal trajectories

## Abstract

**Background:**

Although regular exercise is widely recommended to delay functional decline, few studies have examined the joint associations of exercise frequency and type with trajectories of daily activity limitations among older adults in the UK. This study examined how exercise frequency, type, and their combination associated with population-average trajectories of daily activity limitations in UK older adults.

**Methods:**

We used data from the UK Household Longitudinal Study, including 28,419 person-wave observations of people aged 65 years and older followed across four waves between 2015 and 2023. Exercise frequency and type were assessed at baseline, and the exercise type was categorized as aerobic, resistance/strength, mind-body/balance, or no exercise, with further classification by participation frequency. Daily activity limitations were modelled as count outcomes using negative binomial generalized estimating equations to estimate population-average trajectories over time. Statistical significance was set at *p* < 0.05 (two-sided).

**Results:**

Compared with no exercise, participation in moderate exercise frequency was associated with a 59.1% lower rate of activities of daily living (ADL) limitations (IRR = 0.409, 95% CI: 0.355–0.472, *P* < 0.001) and a 59.4% lower rate of instrumental activities of daily living (IADL) limitations (IRR = 0.406, 95% CI: 0.357–0.462, *P* < 0.001). Aerobic exercise showed the lowest predicted limitation counts over time, followed by mind-body/balance activities. Strength-based exercise demonstrated a frequency-dependent pattern: moderate-frequency participation was associated with 62.0% lower rate of ADL limitations (IRR = 0.380, 95% CI: 0.241–0.601, *P* < 0.001), whereas high-frequency participation did not confer additional benefit (IRR = 1.086, 95% CI: 0.824–1.430, *P* = 0.560) and showed trajectories similar to those of non-exercisers.

**Conclusions:**

This study shows that exercise type and frequency jointly shape population-average trajectories of daily activity limitations in older adults. Aerobic and mind-body/balance exercises are associated with favourable functional trajectories. For resistance/strength training, moderate rather than high participation frequency appears to be associated with better functional outcomes. These findings suggest that both exercise type and frequency may be important considerations when developing exercise guidance to support functional independence in later life.

**Supplementary Information:**

The online version contains supplementary material available at https://doi.org/10.1186/s12916-026-05058-6.

## Background

A rapid increase in the prevalence of functional limitations has been observed in ageing populations, posing major challenges for individuals, health systems, and social care provision. In Europe, recent studies estimate that between 11% and 44% of older adults experience difficulty in at least one basic activity of daily living (ADL), while 8% to 40% face limitations in instrumental activities of daily living (IADLs) [1]. ADLs involve basic self-care activities necessary for independent living, such as bathing and dressing [2]. IADLs include more advanced tasks that require greater cognitive and physical capacity, such as cooking and managing finances [3]. Limitations in ADLs and IADLs serve as key indicators of functional health in older adults and are strongly associated with reduced quality of life [4], increased risk of frailty and disability [5, 6], and mortality [7]. Importantly, the management and care for people with functional limitations carry substantial individual and societal costs in a form of caregiver burden, physician referrals, and institutionalization [8]. Therefore, identifying and addressing the factors that contribute to limitations in ADLs and IADLs among at-risk populations is essential for informing effective policy development and clinical interventions.

Regular physical exercise is widely considered as a key strategy for maintaining physiological functions among older adults and slowing functional decline [9]. From a functional ageing perspective, exercise may help preserve physiological reserve and delay transitions from impairment to disability by maintaining integrated physical, cognitive, and sensorimotor capacity. Substantial evidence supports the beneficial role of exercise in lowering the risk of ADLs and IADLs limitations in older adults [10–12]. For example, aerobic exercise effectively improves cardiorespiratory endurance and overall functional capacity [13], while strength training has been shown to significantly increase muscle strength, enhance physical function, and mitigate sarcopenia and frailty [14]. In addition, mind-body or balance exercises such as Tai Chi, which aim to improve joint flexibility and sensorimotor function, are important for fall prevention and maintaining functional independence by improving proprioception, core strength, and balance in older adults [15].

Despite broad consensus that physical activity is beneficial for healthy ageing, substantial uncertainty remains regarding how exercise type and frequency should be combined to optimally preserve functional independence in later life. Different types of exercise, such as aerobic, strength, and balance training, influence functional health through distinct physiological and neural mechanisms. Aerobic exercise upregulates brain-derived neurotrophic factor (BDNF), supporting neuroplasticity and cognitive function, which is particularly relevant for performing complex IADLs tasks [16]. Balance and neuromuscular exercise help reduce fall risk by improving sensory integration, postural control, and reaction speed [15]. Strength training primarily counteracts muscle loss by activating the mechanistic target of rapamycin (mTOR) pathway and promoting muscle protein synthesis, thereby increasing muscle mass and strength [17]. Current public health guidelines generally promote regular exercise for older adults, yet provide limited empirical guidance on the frequency thresholds at which benefits plateau or potentially diminish, particularly for strength-based exercise. Evidence on strength/resistance training’s association with long-term functional trajectories is limited, and some studies suggest heterogeneous effects depending on frequency, baseline health, and outcomes [18, 19]. In addition, although higher exercise frequency is generally considered beneficial, some studies indicate that for certain exercise modalities, especially high-intensity or strength-based training, excessively frequent sessions may pose risks, especially for older population. These include insufficient recovery, accumulated micro-injuries, or chronic inflammation due to overtraining, which could attenuate protective effects or even lead to adverse outcomes [20]. For instance, a meta-analysis in patients with Alzheimer’s disease suggested that moderate-frequency (4–5 times per week) aerobic exercise yielded the most significant improvement in ADLs [21]. Consequently, it remains unclear whether higher frequency participation consistently confers additional protection against functional decline, or whether moderate engagement may represent a more sustainable and effective strategy for maintaining activities of daily living in older adulthood.

To address these gaps mentioned above, this study aimed to examine the independent and joint associations of exercise frequency and type with population-average trajectories of daily activity limitations in older adults in the UK, with particular attention to whether different exercise modalities exhibit frequency-dependent associations with functional decline.

Using longitudinal data from the UK Household Longitudinal Study (UKHLS), this study focuses on individuals aged 65 years and older. We hypothesized that both the type and frequency of exercise at baseline independently and interactively associated with population-average trajectories of functional limitations in older adulthood. Specifically, we proposed three a priori hypotheses:

H1: Among adults aged 65 years and older, the number of ADL and IADL limitations increases over time, and baseline exercise type and frequency are associated with differences in the rate of functional decline over follow-up.

H2: The accumulation of ADL and IADL limitations is slower among older adults who participate in aerobic, resistance/strength, or mind-body/balance exercises than among those reporting no exercise.

H3: There is a frequency-dependent effect of strength-based exercise on the time trend of ADLs/IADLs limitations counts, with moderate-frequency participation associated with the lowest predicted limitation counts over time while the high-frequency group no longer demonstrates additional benefits.

## Methods

### Data source

This study used data from the UK Household Longitudinal Study, also known as Understanding Society [22]. Starting in 2009, the UKHLS is an ongoing, large-scale, multi-stage, probability-based household panel survey designed to capture comprehensive longitudinal data on health, social, and economic dynamics and stability annually among all four countries of the UK population. We used data from waves 5 (2013–2015), 7 (2015–2017), 9 (2017–2019), 11 (2019–2021), and 13 (2021–2023) of UKHLS. Wave 5 data were utilized exclusively to establish baseline exposures (exercise) and prior sociodemographic covariates. This established the temporal precedence required before the prospective outcome assessment period, which was tracked at waves 7, 9, 11, and 13. There were 8,609 participants aged 65 years and older at wave 7 (28,419 person-wave observations from wave 7 to wave 13). Inclusion criteria were defined as individuals aged 65 years and older who successfully participated in the wave 7 survey and had available outcome data during follow-up. Exclusion criteria consisted of missing data on baseline ADL or IADL measures in wave 7, or missing survey responses for the specified exercise exposures and primary sociodemographic covariates in wave 5. We excluded 4,298 observation points with missing data on ADLs or IADLs, and 129 participants with missing information on exposures or covariates. As there were less than 1% missing in exposure/covariates, a complete case analysis was conducted. 23,992 observation points (8,186 participants at baseline) were included in this study.

### Exposure: exercise

Exercise-related variables were collected at wave 5. We examined three exercise measures: exercise frequency, exercise type, and a combined measure incorporating both frequency and type. Exercise frequency was derived from the UKHLS questionnaire, which asked participants how often in the past 12 months they engaged in their primary exercise. Based on the original survey response options, we categorized participants into four mutually exclusive frequency groups: no exercise; low frequency (less often than once per week but at least once a year); moderate frequency (at least once per week but fewer than three times per week); and high frequency (three or more times per week) [23, 24]. Type of exercise was classified into four mutually exclusive categories: no exercise, aerobic, resistance/strength, or mind-body/balance and others. Exercise types were grouped according to their physiological characteristics, informed by prior literature and public health guidance [25, 26]. The detailed classification scheme is provided in Additional file 1: Table 1. Combined exercise frequency and type was constructed by cross-classifying the primary reported exercise modality with the mutually exclusive frequency tiers, resulting in ten categories: no exercise, high frequency resistance/strength, moderate frequency resistance/strength, low frequency resistance/strength, high frequency mind-body/balance and others, moderate frequency mind-body/balance and others, low frequency mind-body/balance and others, high frequency aerobic, moderate frequency aerobic, low frequency aerobic. As this is an observational study utilising survey data rather than a controlled clinical trial, exercise consisted of self-directed, free-living activities rather than professionally monitored training programs.

### Outcome: daily activity limitations

Limitations in daily functioning were assessed using self-reported ADLs and IADLs at waves 7, 9, 11, and 13, following the UKHLS social care module [27]. Assessments were conducted during the biennial UKHLS data collection waves, resulting in approximately two-year intervals between measurements. ADLs limitations were defined as counting the number of activities for which participants reported any difficulty in manage stairs, get around house, get in/out of bed, cut toenails, bath/shower, use toilet, eat, wash face and hands, get dressed and undressed, or take the right number of medicines at the right times. IADLs limitations were defined as counting the number of activities for which participants reported any difficulty in walk down road, do the shopping, do housework or laundry, or do paperwork or pay bills. These ADLs and IADLs measures are widely used indicators of functional health in ageing research and have demonstrated acceptable validity and reliability in previous population-based studies of older adults [28].

### Covariates

Potential confounders were selected based on previous literature and their availability in the dataset. Covariates measured at wave 5 included age, sex, highest educational qualification, ethnicity, partnership status, urban or rural residence, household composition, and long-standing illness/disability (long-standing physical or mental impairment, illness, or disability over a period of at least 12 months). In sensitivity analyses, additional adjustment was made for mental health conditions and health-related behaviours, including smoking status and alcohol consumption frequency. These variables were considered potential confounders or mediators and were therefore included only in sensitivity analyses to avoid over-adjustment in primary models.

### Statistical analysis

We used generalized estimating equation (GEE) procedure to build the negative binomial models to assess the association between exercise frequency, type, type consider frequency and ADLs/IADLs limitations trajectories. GEE models were selected because the primary objective was to estimate population-average longitudinal associations while accounting for within-individual correlation across repeated observations. Given the count nature of the outcomes and evidence of overdispersion, negative binomial models with a log link were specified [29]. An exchangeable working correlation structure was used to account for within-individual correlation across repeated measurements, and robust standard errors were obtained to ensure valid inference under potential misspecification of the correlation structure. Model goodness of fit was assessed using the quasi-likelihood under the independence model criterion, with lower values indicating better fit. All models were adjusted for baseline age, sex, educational attainment, ethnicity, partnership status, urbanicity, household composition, and long-standing illness or disability. Time since baseline was modelled as a continuous variable. Quadratic terms for time were not included in the primary models, as interaction terms between time and exercise exposures were statistically significant only in ADLs models (see Additional file 1: Table 2). However, interactions between time and exercise exposures were retained in all final models to formally test whether exercise frequency or type was associated with differential rates of change in functional limitations over follow-up.

To reduce potential reverse causation, we conducted sensitivity analyses excluding 2,951 participants who reported any ADLs or IADLs limitation at wave 7, as functional limitations may restrict participation in exercise. Additional sensitivity analyses further adjusted for mental health conditions and health behaviours. We also fitted random-effects logistic regression models for dichotomized ADLs and IADLs outcomes to assess the robustness of findings across modelling approaches.

All analyses were conducted in Stata version 19.0 (Stata Corp., College Station, Texas 77845, USA). Statistical tests were two-sided with a significance level of *p* < 0.05.

## Results

The analytic sample comprised 8,186 participants aged 65 years and older at baseline. The mean age was 74.0 years (SD 6.90), and 54.5% of participants were women. At baseline, the mean number of ADLs limitations was 0.52 (SD 1.25), and the mean number of IADLs limitations was 0.54 (SD 1.08). At wave 5, 40.8% of participants reported no regular exercise, while 28.2% reported low-frequency exercise, 23.0% reported moderate-frequency exercise, and 8.1% reported high-frequency exercise. Aerobic exercise was the most commonly reported main type of exercise (33.9%), followed by mind-body/balance and other activities (20.8%) and resistance/strength exercise (4.5%). Detailed baseline characteristics of the study population are presented in Table 1.

**Table 1:** Baseline characteristics of participants

Characteristics	*n* = 8,186
**ADLs limitations**,** Mean (SD)**^**a**^	0.52 (1.25)
**IADLs limitations**,** Mean (SD)**^**a**^	0.54 (1.08)
**Exercise frequency**,** N (%)**^**b**^	
* No exercise*	3,341 (40.81)
* Low*	2,305 (28.16)
* Moderate*	1,880 (22.97)
* High*	660 (8.06)
**Main type of exercise**,** N (%)**^**b**^	
* No exercise*	3,341 (40.81)
* Aerobic*	2,772 (33.86)
* Resistance/strength*	370 (4.53)
* Mind-body/balance and others*	1,703 (20.80)
**Age (years)**,** Mean (SD)**^**a**^	73.97 (6.86)
**Sex**,** N (%)**^**b**^	
* Male*	3,728 (45.54)
* Female*	4,458 (54.46)
**Highest qualification**,** N (%)**^**b**^	
* Degree/professional or higher (higher than A-levels or equivalent)*	2,214 (27.05)
* School level (up to A-levels or equivalent)*	3,634 (44.39)
* No qualification*	2,338 (28.56)
**Ethnicity**,** N (%)**^**b**^	
* White*	7,760 (94.80)
* Others*	426 (5.20)
**Partnership status**,** N (%)**^**b**^	
* Married or cohabitation*	5,298 (64.72)
* Others*	2,888 (35.28)
**Urbanity**,** N (%)**^**b**^	
* Urban*	5,562 (67.95)
* Rural*	2,624 (32.06)
**Composition of household**,** N (%)**^**b**^	
* Living alone*	2,494 (30.47)
* Living with others*	5,692 (69.53)
**Have long-standing illness or disability**,** N (%)**^**b**^	
* No*	3,684 (45.00)
* Yes*	4,502 (55.00)

Abbreviation: *ADLs* activities of daily living, *IADLs *instrumental activities of daily living, *N* sample size, *SD* standard deviation

^a^Data from wave 7(2015–2017)

^b^Data from wave 5 (2013–2015)

**Fig. 1:**
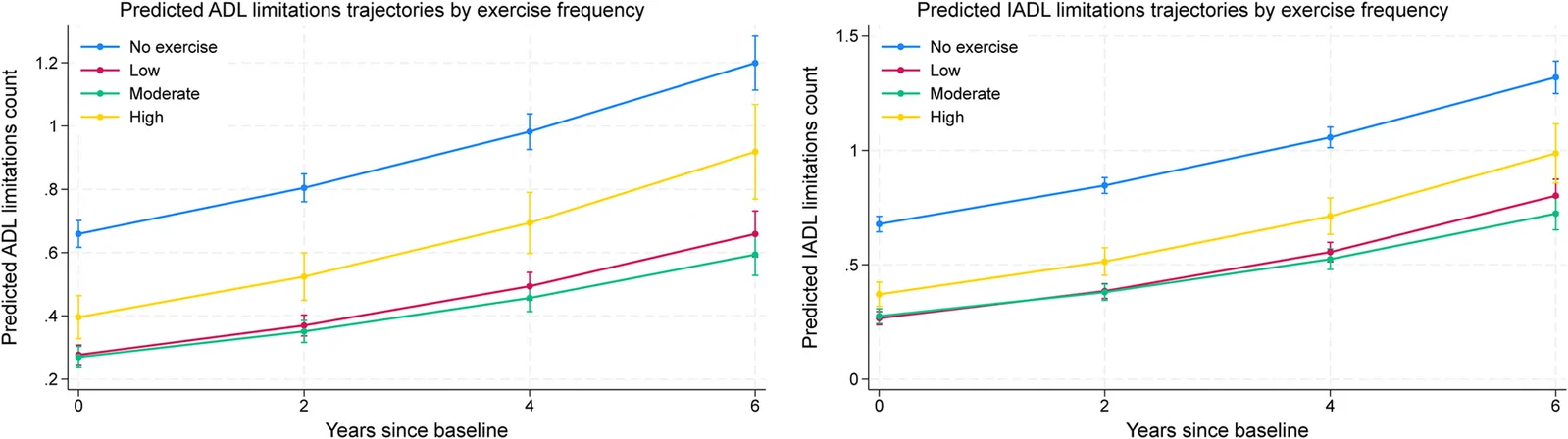
Predicted daily activity limitations by exercise frequency

**Fig. 2:**
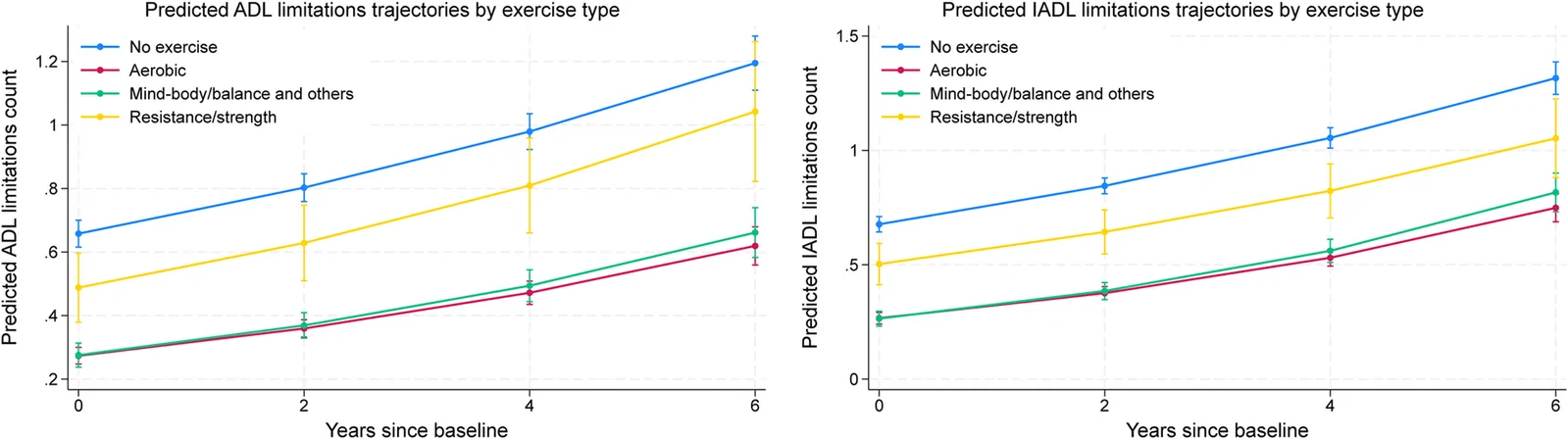
Predicted daily activity limitations by exercise type

**Fig. 3:**
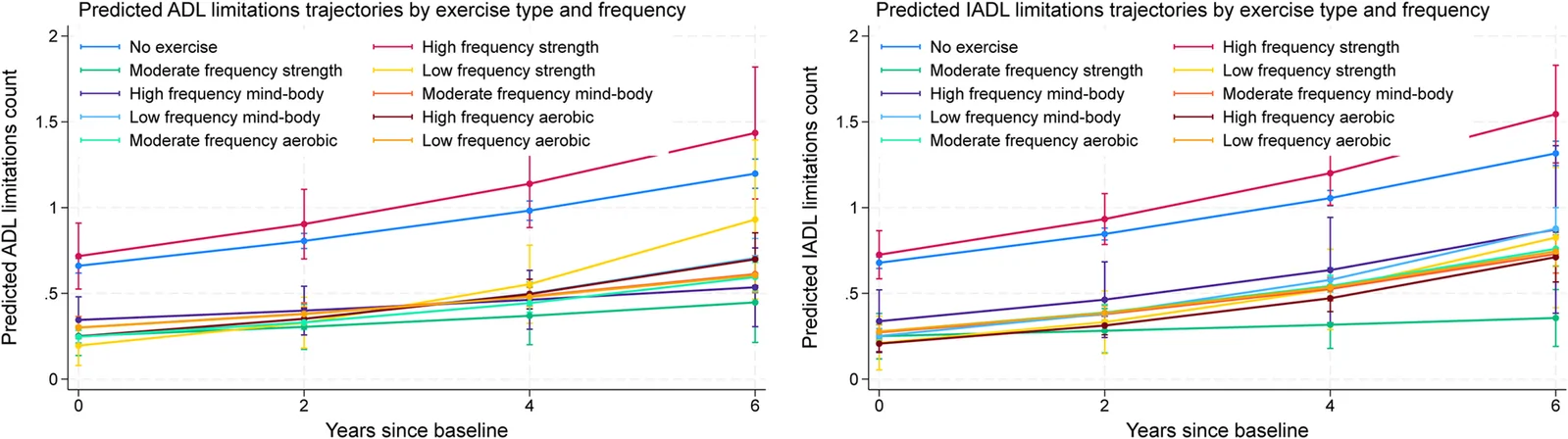
Predicted daily activity limitations by exercise type and frequency

The associations between exercise frequency, exercise type, and combined exercise type and frequency with ADL and IADL limitations are presented in Table 2; Figs. 1, 2 and 3, and the modelling procedure are shown in Additional file 1: Table 2.

Compared with no exercise participation, low, moderate, and high exercise frequency were each associated with fewer ADL limitations over follow-up. The interaction between time since baseline and exercise frequency indicated that differences in ADL trajectories widened across waves. Predicted ADL counts increased over time in all groups, but the participants reporting moderate-frequency baseline exercise maintained the lowest predicted limitation counts throughout follow-up. A similar pattern was observed for IADL limitations, with participants engaging in any level of exercise maintaining consistently lower predicted IADL difficulty counts throughout follow-up. The magnitude and direction of associations for IADLs were broadly comparable to those observed for ADLs (Fig. 1). In Table 2, we can see that compared with no exercise, low-frequency exercise was associated with a 58.0% lower rate of ADL limitations (IRR = 0.420, 95% CI: 0.367–0.480, *P* < 0.001) and a 60.7% lower rate of IADL limitations (IRR = 0.393, 95% CI: 0.348–0.444, *P* < 0.001). Moderate-frequency exercise showed a 59.1% lower rate of ADL limitations (IRR = 0.409, 95% CI: 0.355–0.472, *P* < 0.001) and a 59.4% lower rate of IADL limitations (IRR = 0.406, 95% CI: 0.357–0.462, *P* < 0.001). High-frequency exercise was associated with a 39.9% lower rate of ADL limitations (IRR = 0.601, 95% CI: 0.499–0.722, *P* < 0.001) and a 45.3% lower rate of IADL limitations (IRR = 0.547, 95% CI: 0.470–0.637, *P* < 0.001). The interaction between time and exercise frequency was significant for ADL (*P* = 0.002) and IADL (*P* < 0.001).

**Table 2:** Associations between exercise and daily activity limitations in population-average negative binomial GEE models

	IRR	95% CI	*P* value	*P* value for interaction	IRR	95% CI	*P* value	*P* value for interaction
**Panel A:**								
Data collection time (wave)	1.105	1.090 to 1.120	<0.001		1.117	1.105 to 1.130	<0.001	
Exercise frequency (ref: no exercise)								
* Low*	0.420	0.367 to 0.480	<0.001		0.393	0.348 to 0.444	<0.001	
* Moderate*	0.409	0.355 to 0.472	<0.001		0.406	0.357 to 0.462	<0.001	
* High*	0.601	0.499 to 0.722	<0.001		0.547	0.470 to 0.637	<0.001	
Wave * exercise frequency (ref: no exercise)				0.002				<0.001
* Low*	1.046	1.018 to 1.074	0.001		1.075	1.051 to 1.100	<0.001	
* Moderate*	1.032	1.005 to 1.060	0.021		1.051	1.027 to 1.076	<0.001	
* High*	1.041	1.006 to 1.078	0.021		1.054	1.023 to 1.085	<0.001	
**Panel B**:								
Data collection time (wave)	1.105	1.090 to 1.120	<0.001		1.117	1.105 to 1.129	<0.001	
Main type of exercise (ref: no exercise)								
* Aerobic*	0.416	0.370 to 0.468	<0.001		0.394	0.353 to 0.440	<0.001	
* Mind-body/balance and others*	0.419	0.359 to 0.489	<0.001		0.391	0.342 to 0.447	<0.001	
* Resistance/strength*	0.742	0.587 to 0.938	0.013		0.743	0.617 to 0.895	0.002	
Wave * exercise type (ref: no exercise)				0.002				<0.001
* Aerobic*	1.037	1.012 to 1.063	0.003		1.063	1.041 to 1.086	<0.001	
* Mind-body/balance and others*	1.048	1.017 to 1.078	0.002		1.080	1.053 to 1.107	<0.001	
* Resistance/strength*	1.027	0.986 to 1.071	0.202		1.012	0.980 to 1.046	0.465	
**Panel C**:								
Data collection time (wave)	1.104	1.089 to 1.119	<0.001		1.117	1.105 to 1.129	<0.001	
Exercise type and frequency (ref: no exercise)								
* High frequency resistance/strength*	1.086	0.824 to 1.430	0.560		1.069	0.876 to 1.304	0.511	
* Moderate frequency resistance/strength*	0.380	0.241 to 0.601	<0.001		0.370	0.218 to 0.628	<0.001	
* Low frequency resistance/strength*	0.296	0.163 to 0.539	<0.001		0.310	0.147 to 0.654	0.002	
* High frequency mind-body/balance and others*	0.523	0.352 to 0.776	0.001		0.498	0.289 to 0.858	0.012	
* Moderate frequency mind-body/balance and others*	0.455	0.363 to 0.571	<0.001		0.402	0.334 to 0.484	<0.001	
* Low frequency mind-body/balance and others*	0.375	0.307 to 0.458	<0.001		0.371	0.309 to 0.445	<0.001	
* High frequency aerobic*	0.377	0.299 to 0.476	<0.001		0.305	0.242 to 0.385	<0.001	
* Moderate frequency aerobic*	0.374	0.317 to 0.441	<0.001		0.409	0.346 to 0.484	<0.001	
* Low frequency aerobic*	0.454	0.388 to 0.533	<0.001		0.409	0.353 to 0.475	<0.001	
Wave * exercise type and frequency (ref: no exercise)				<0.001				<0.001
* High frequency resistance/strength*	1.017	0.964 to 1.073	0.548		1.016	0.977 to 1.055	0.426	
* Moderate frequency resistance/strength*	0.997	0.927 to 1.072	0.932		0.949	0.879 to 1.025	0.186	
* Low frequency resistance/strength*	1.174	1.042 to 1.324	0.009		1.124	0.981 to 1.289	0.092	
* High frequency mind-body/balance and others*	0.974	0.910 to 1.043	0.455		1.049	0.953 to 1.155	0.330	
* Moderate frequency mind-body/balance and others*	1.019	0.977 to 1.064	0.381		1.055	1.019 to 1.092	0.002	
* Low frequency mind-body/balance and others*	1.078	1.040 to 1.118	<0.001		1.103	1.067 to 1.140	<0.001	
* High frequency aerobic*	1.075	1.023 to 1.130	0.004		1.100	1.049 to 1.154	<0.001	
* Moderate frequency aerobic*	1.048	1.016 to 1.082	0.004		1.059	1.027 to 1.093	<0.001	
* Low frequency aerobic*	1.018	0.984 to 1.054	0.308		1.055	1.026 to 1.086	<0.001	

Adjusted for baseline age, sex, educational attainment, ethnicity, partnership status, urbanicity, household composition, and long-standing illness/disability

IRR: incidence rate ratio; CI: confidence interval; ref: reference

Note: P value less than 0.05 would be considered as statistically significant. Covariates’ coefficients are not shown for the sake of brevity

When exercise type was examined, aerobic activities were associated with the lowest predicted ADL and IADL limitation counts across follow-up, compared with no exercise. Mind-body/balance and other activities showed a similar, though slightly weaker, protective pattern. Strength-related activities were also associated with fewer limitations relative to no exercise, but the magnitude of association was smaller than that observed for aerobic activities for both ADLs and IADLs (Fig. 2). Compared with no exercise, aerobic exercise was associated with a 58.4% lower rate of ADL limitations (IRR = 0.416, 95% CI: 0.370–0.468, *P* < 0.001) and a 60.6% lower rate of IADL limitations (IRR = 0.394, 95% CI: 0.353–0.440, *P* < 0.001). Mind-body/balance exercise showed similar reductions (ADL: IRR = 0.419, 95% CI: 0.359–0.489, *P* < 0.001; IADL: IRR = 0.391, 95% CI: 0.342–0.447, *P* < 0.001). Resistance/strength exercise was associated with more modest reductions (ADL: IRR = 0.742, 95% CI: 0.587–0.938, *P* = 0.013; IADL: IRR = 0.743, 95% CI: 0.617–0.895, *P* = 0.002) (Table 2).

Results for combined type-frequency categories demonstrated greater heterogeneity in functional trajectories (Fig. 3). People with high-frequency resistance/strength activities showed the highest burden of ADL and IADL limitations. Participants reporting no exercise showed a progressively increasing trajectory, with higher predicted ADL and IADL limitation counts at every time point compared with those engaging in aerobic or mind-body/balance activities. Aerobic and mind-body/balance activities, particularly at low to moderate frequencies, were associated with relatively flatter trajectories, suggesting slower accumulation of functional limitations over time. High-frequency strength-related activities were associated with higher predicted ADL and IADL limitation counts compared with moderate-frequency resistance/strength activities, which showed a less steep increase during follow-up. The widening separation of trajectories over time indicates that differences in exercise engagement frequency and type at baseline were associated with progressively larger differences in functional health. Moderate-frequency resistance/strength training was associated with a 62.0% lower rate of ADL limitations (IRR = 0.380, 95% CI: 0.241–0.601, *P* < 0.001) and a 63.0% lower rate of IADL limitations (IRR = 0.370, 95% CI: 0.218–0.628, *P* < 0.001). In contrast, high-frequency resistance/strength training showed no significant benefit for ADL (IRR = 1.086, 95% CI: 0.824–1.430, *P* = 0.560) or for IADL (IRR = 1.069, 95% CI: 0.876–1.304, *P* = 0.511). High-frequency aerobic exercise remained beneficial (ADL: IRR = 0.377, 95% CI: 0.299–0.476, *P* < 0.001), but the effect size was similar to moderate-frequency aerobic exercise. The overall interaction between time and combined type-frequency was significant for both ADL and IADL (*P* < 0.001) (Table 2).

The strength and magnitude of associations were largely unchanged across alternative analytical assumptions and model specifications. When participants with any ADL or IADL limitation at baseline were excluded to reduce potential reverse causation (*n* = 2,951), the associations between exercise frequency, type, and subsequent ADL and IADL limitations remained consistent with the primary analyses (see Additional file 1: Table 3). Additional adjustment for mental health conditions, drinking frequency, and smoking status did not materially alter effect estimates, although confidence intervals were modestly attenuated for some exposure categories (see Additional file 1: Table 4). Furthermore, random-effects logistic regression models using dichotomized outcomes, defined as the presence of any ADL or IADL limitation, produced estimates that were consistent in direction and statistical significance with those from the population-average GEE models (see Additional file 1: Table 5).

## Discussion

### Main findings

This longitudinal study utilized data from the UKHLS to investigate the associations between exercise frequency, exercise type, and their combination with trajectories of daily activity limitations in older adults. The results demonstrated that exercising at a moderate frequency of one to two times per week was associated with more favourable population-average functional trajectories over follow-up. Regarding exercise type, aerobic exercise was linked to the lowest cumulative burden of ADLs and IADLs limitations, and this was followed by mind-body/balance exercises. In contrast, resistance/strength training exhibited a comparatively weaker protective effect. Notably, high-frequency resistance/strength training, defined as three or more sessions per week, was related to a greater burden of functional limitations, a level approaching that observed among non-exercisers. This suggests that high-frequency resistance/strength participation was not associated with additional functional benefit in this population, and this pattern may reflect heterogeneity in health status, exercise intensity, recovery capacity, or measurement limitations. The magnitude of associations was substantial across exercise categories, with IRRs generally indicating approximately 40%-60% lower rates of functional limitations compared with non-exercisers. The effect sizes demonstrate substantial clinical relevance, for instance, moderate-frequency aerobic exercise was associated with an approximately 60% reduction in the incidence rate of functional limitations over the follow-up period, representing a highly meaningful preservation of independence. Overall, the findings were broadly consistent with our a priori hypotheses. Specifically, the findings were consistent with H1, as ADL and IADL limitations increased over time and baseline exercise frequency and type were associated with differential rates of functional decline. The findings supported H2, because participants engaging in any type of exercise, including aerobic, mind-body/balance, or resistance/strength, showed slower accumulation of limitations compared with the no exercise group. The findings were consistent with H3, as resistance/strength training exhibited a clear frequency-dependent pattern: moderate frequency was associated with the most favourable trajectories, whereas high frequency was not associated with greater benefit in this study population.

### Comparison with previous studies and potential mechanisms

Previous research has examined associations between specific exercise types and functional health in older adults, with inconsistent conclusions and a predominant focus on single modalities. For instance, dance has been linked to reduced risk of ADL disability [30], while aerobic exercise may protect against IADLs decline [31]. Such studies often consider each exercise type holistically, suggesting that distinct physiological mechanisms, such as cognitive-motor integration in dance or enhanced flexibility in aerobics, underlie their benefits. However, the role of participation frequency within each modality and its joint effects with exercise type remains underexplored. For instance, evidence shown that community-dwelling older adults engaging in aerobic, strengthening, and balance activities had better physical function than non-participants [32], but it did not examine how the frequency of each exercise type contributed to these outcomes. Similarly, a meta-analysis reported that moderate-to-high physical activity reduced ADL disability risk by approximately 49% [33], yet it pooled different exercise types and frequencies without assessing their joint effects. Our findings extend this evidence by confirming the significance of exercise type and demonstrating that moderate frequency aerobic exercise offers the strongest protection against cumulative ADLs and IADLs burden, followed by mind-body/balance exercises. While well-designed, progressive resistance training under professional supervision is known to enhance dynamic regulation, the self-reported, free-living strength activities captured in this older population survey may vary widely in execution, potentially diluting the observed cognitive-motor benefits compared to clinical trials.

The distinct protective effects of exercise types against functional limitations arise from divergent physiological pathways, clarifying the observed hierarchy in our findings. Aerobic exercise improves cardiorespiratory fitness and upregulates BDNF [34], thereby supporting neuroplasticity and executive function, key determinants of performance in cognitively demanding IADLs. Mind-body/balance training enhance proprioception, multisensory integration, and postural control [35], directly mitigating fall risk and preserving mobility-dependent ADLs [36]. Moreover, both aerobic and mind-body/balance exercises have been shown to reduce depressive symptoms and improve self‑efficacy, psychological pathways that can further facilitate the performance of complex daily tasks [37, 38]. In contrast, although strength training robustly increases muscle mass and strength via mTOR-mediated protein synthesis, effectively countering sarcopenia, some forms of traditional resistance-focused training may provide less direct stimulation of integrated cognitive-motor and postural control processes than multimodal aerobic or balance-oriented activities [39, 40], while appropriately designed strength training programs can improve these functions. Consequently, modalities that concurrently engage cardiovascular, neural, and sensorimotor systems, such as aerobic and balance exercise, deliver broader functional protection, consistent with the multidimensional demands of both ADLs and IADLs.

While existing recommendations emphasize regular participation in exercise, our results suggest that the functional benefits of exercise depend not only on participation but also on the alignment between exercise type and frequency. Resistance/strength training demonstrated a distinct frequency-dependent pattern: while moderate frequency was linked to favourable functional trajectories, higher frequency was not associated with further protection and, in some models, approached the trajectory observed in non-exercisers. The evidence on occupational physical activity similarly observed that higher levels of physical activity are not universally beneficial, a concept termed the “physical activity paradox” [41], and our results extend this paradox to resistance/strength exercise in older adults. The observed frequency-dependent plateau in resistance training may be explained by the physiological requirement for extended recovery intervals in older adults. Adequate recovery is necessary to facilitate optimal muscle protein synthesis and mitigate acute systemic inflammatory responses following mechanical loading. One possible explanation is that older adults may require longer recovery periods between resistance training sessions because ageing is associated with slower physiological recovery following exercise. However, these mechanisms were not measured in the present study. Insufficient recovery between sessions could promote cumulative microtrauma and low-grade inflammation, elevating circulating pro‑inflammatory cytokines such as interleukin‑6 and C‑reactive protein [42], potentially attenuating the anabolic adaptations mediated through pathways such as mTOR signalling [43]. Alternative explanations include residual confounding, heterogeneity in exercise intensity and training quality, or health selection processes among participants engaging in high-frequency resistance training. Therefore, these findings should be interpreted cautiously and require confirmation in studies using objective measures of exercise dose and training characteristics.

Moderate-frequency exercise may represent a sustainable pattern of engagement that balances physiological stimulation with recovery demands in older adults. In contrast, higher frequencies may not necessarily confer additional benefit, particularly when exercise intensity, duration, and recovery periods are unknown. Behavioural factors, including adherence, enjoyment, fatigue, and long-term sustainability, may also contribute to the observed patterns. Because these factors were not measured in this study, these interpretations should be considered speculative and require further investigation. Moderate-frequency exercise was associated with more favourable functional trajectories over follow-up, likely reflecting an optimal balance between physiological stimulus and recovery [44]. This balance permits sufficient time for tissue repair and functional adaptation, thereby preserving muscular strength, cardiovascular efficiency, and neural integrity, while simultaneously mitigating excessive catabolic stress and low-grade systemic inflammation [45]. Moreover, it supports sustained expression of neurotrophic factors, including BDNF, which underpin cognitive processes essential for performing complex ADLs and IADLs [46]. In contrast, higher frequencies confer no additional functional benefit, with outcomes in some cases being comparable to no exercise. Insufficient recovery intervals may precipitate cumulative microdamage, persistent inflammation, and a net catabolic milieu, undermining adaptive gains in physical function [47]. Excessive frequency further increases the risk of nonfunctional overreaching, systemic fatigue, and elevated cortisol and interleukin-6 levels, adversely affecting both physical performance and behavioural motivation [48, 49]. Persistent fatigue may also lower pain tolerance and increase perceived exertion during daily tasks, further discouraging consistent exercise [50, 51]. Over time, the physiological burden may erode cognitive stamina and reduce adherence, ultimately compromising the consistent engagement required for ADLs and IADLs maintenance. In addition, higher-frequency resistance/strength training may be more physically demanding, less enjoyable, or more difficult to sustain among older adults, potentially leading to fatigue, pain, or reduced adherence over time. Such factors could attenuate functional benefits independent of physiological adaptation and may partially explain why moderate-frequency engagement appeared more favourable in general populations.

### Public health and clinical implications

These findings have important implications for physical activity guidance and intervention design for older adults. First, encouraging regular physical activity in older adults, especially aerobic and mind-body/balance exercises, may help support maintenance of functional independence. Second, exercise recommendations may benefit from considering exercise type and frequency: for resistance/strength training, moderate-frequency resistance training appeared to be associated with more favourable outcomes than high-frequency participation in this study population. The absence of additional benefit associated with high-frequency resistance/strength training highlights the importance of incorporating recovery considerations and recovery considerations into exercise guidance for older populations. Well-designed intervention studies are essential to validate the effects of different exercise prescriptions (including combinations of type, frequency, and intensity) and to provide a scientific foundation for developing precise, personalized, and safe exercise recommendations for older adults.

### Strengths and limitations

This study has several strengths. First, we used repeated measures of ADLs and IADLs collected over multiple waves, allowing us to examine trajectories of functional limitations rather than cross-sectional associations or single transition events. Second, ADLs and IADLs limitations were modelled as count outcomes using population-average negative binomial GEE models, which appropriately account for the distributional characteristics of functional limitation counts and within-person correlation over time. Sensitivity analyses using dichotomized ADL and IADL outcomes and random-effects logistic regression yielded consistent results, supporting the robustness of findings across outcome definitions and modelling strategies. Third, we conducted a series of sensitivity analyses, including exclusion of participants with baseline functional limitations and additional adjustment for mental health conditions and health behaviours, which reduced concerns related to reverse causation and health selection.

However, several limitations should be considered when interpreting these findings. Exercise exposure was self-reported and measured at a single baseline wave, which may be subject to recall bias and social desirability bias, and precluded assessment of changes in exercise behaviour over time. Misclassification of exposure is therefore likely, particularly if participants altered their exercise patterns during follow-up. Participants engaging in multiple exercise activities were categorized according to the exercise type performed most frequently, which does not fully capture the complexity or multidimensional nature of real-world exercise behaviours, thus the true protective effects of exercise could be stronger than observed. Exercise modalities were not mutually exclusive in real-world behaviour, and classification in this study may have obscured mixed-modality exercise patterns. In addition, the exercise only has frequency measures available, but no other key dimensions of exercise collected, such as intensity or duration. The lack of data regarding the physiological intensity or duration of the exercise sessions prevents a comprehensive dose-response analysis and remains a limitation of the survey-based data. Information on non-leisure physical activity, such as occupational activity, active transport, or household work, was not available, which may have led to incomplete characterization of participants’ overall activity profile beyond exercise. The observational nature of the data means that residual confounding by unmeasured or imperfectly measured factors, such as baseline physical performance, subclinical disease, pain, or motivation, cannot be entirely eliminated, and causal inference is limited by the observational study design. Additionally, the finding that high-frequency resistance and strength exercise was not associated with additional functional benefit and in some models approached trajectories observed in non-exercisers should be interpreted with caution. Residual confounding by unmeasured health-related factors such as subclinical disease, pain, pre-existing functional decline, or exercise motivation may partly explain this pattern. For instance, individuals who exercise at high frequency may include those managing existing health conditions or recovering from injuries, which could attenuate or mask a true protective effect. Furthermore, the absence of information on exercise intensity and session duration limits our ability to fully characterise the dose-response relationship. High-frequency sessions of low intensity may differ substantially in physiological effect from high-frequency high-intensity training. Therefore, the observed findings regarding high-frequency resistance/strength exercise should not be interpreted as evidence that such exercise is harmful in older adults. The use of a complete-case analysis may introduce selection bias if individuals with missing data have systematically different functional trajectories or lower baseline exercise participation rates than the analytical sample. However, the missing rate in outcomes was less than 5%, and sensitivity analyses excluding participants with baseline functional limitations yielded consistent results. People exercising moderately may represent sustainable habitual exercisers, have balanced health status, avoid both frailty and excessive training burden, while high-frequency groups may include people exercising because of rehabilitation or illness management. Residual health selection may remain even after excluding participants with baseline functional limitations, as subclinical disease, pain, frailty, or declining physical capacity may influence both exercise participation and later functional outcomes. Attrition and non-response over follow-up may also introduce selection bias, as participants with worse health or more severe functional limitations are more likely to drop out, potentially leading to underestimation of associations. ADL and IADL limitations were self-reported and may be influenced by reporting differences related to expectations, coping strategies, or social context. Although these measures are widely used and validated in previous studies, they do not capture performance-based functional capacity and may be less sensitive to early functional decline.

## Conclusions

While these results are observational, they suggest that exercise type and frequency are jointly associated with the developmental trajectory of daily activity limitations in older adults. Specifically, aerobic exercise demonstrates the strongest protective effect against functional decline, followed by mind-body/balance exercise. In contrast, resistance/strength training exhibits clear frequency dependence: participants engaging in moderate-frequency training maintained the lowest predicted limitation counts throughout follow-up, whereas higher-frequency training is associated with no additional benefit and corresponds to trajectories comparable to those of the inactive group. These findings suggest that moderate-frequency resistance training may be preferable to high-frequency participation in general population settings, although this finding requires confirmation in studies with objective measures of exercise dose and training characteristics. These findings may help inform future intervention studies and exercise guidance by suggesting that both exercise type and frequency warrant consideration when supporting functional independence in older adults.

## Supplementary Information

Below is the link to the electronic supplementary material.

Supplementary Material 1: Additional file 1: Supplementary Tables 1–5.

## Data Availability

The dataset, UK Household Longitudinal Study (Understanding Society), supporting the findings of this article is available for download from the UK Data Service (https://www.ukdataservice.ac.uk) and can be used after registration and acceptance of the UK Data Service end user licence: “Understanding Society: Waves 1-15, 2009-2024 and Harmonised BHPS: Waves 1-18, 1991-2009” (https://datacatalogue.ukdataservice.ac.uk/studies/study/6614).

## Abbreviations

ADLs: activities of daily living
IADLs: instrumental activities of daily living
BDNF: brain-derived neurotrophic factor
mTOR: mechanistic target of rapamycin
UKHLS: UK Household Longitudinal Study
GEE: generalized estimating equation
